# Effect of head position changes on the depth of tracheal intubation in pediatric patients: A prospective, observational study

**DOI:** 10.3389/fped.2022.998294

**Published:** 2022-09-08

**Authors:** Peier Zhuang, Weikai Wang, Minghua Cheng

**Affiliations:** Department of Anesthesiology, The First Affiliated Hospital of Shantou University Medical College, Shantou, China

**Keywords:** pediatric, head position, tracheal intubation, endotracheal tube depth, displacement, intubation complications

## Abstract

**Purpose:**

The purpose of this study was to investigate the effect of changing head position on the endotracheal tube (ETT) depth and to assess the risk of inadvertent extubation and bronchial intubation in pediatric patients.

**Methods:**

Subjects aged 4–12 years old with orotracheal intubation undergoing elective surgeries were enrolled. After induction, the distances between “the ETT tip and the trachea carina” (T-C) were measured using a Disposcope flexible endoscope in head neutral position, 45° extension and flexion, 60° right and left rotation. The distance of the ETT tip movement relative to the neutral position (ΔT-C) was calculated after changing the head positions. The direction of the ETT tip displacement and the adverse events including endobronchial intubation, accidental tracheal extubation, hoarseness and sore throat were recorded.

**Results:**

The ETT tip moved toward the carina by 0.5 ± 0.4 cm (*P* < 0.001) when the head was flexed. After extending the head, the ETT tip moved toward the vocal cord by 0.9 ± 0.4 cm (*P* < 0.001). Right rotation resulted that the ETT tip moved toward the vocal cord direction by 0.6 ± 0.4 cm (*P* < 0.001). Moreover, there was no displacement with the head on left rotation (*P* = 0.126). Subjects with the reinforced ETT had less ETT displacement after changing head position than the taper guard ETT.

**Conclusion:**

The changes of head position can influence the depth of the ETT especially in head extension. We recommend using the reinforced ETT to reduce the ETT displacement in pediatrics to avoid intubation complications.

**Clinical trial registration:**

[www.ClinicalTrials.gov], identifier, [ChiCTR2100042648].

## Introduction

Children have a short tracheal length with a small margin of safety regarding correct endotracheal tube (ETT) placement (1). Inappropriate placement of the ETT will increase the risk of inadvertent extubation and bronchial intubation (2). Matsuoka et al. (3) suggested that the occurrence of the improper ETT depth during PICU was 23.5%. Unintentional extubation was the most common intubation complication among infants and children in the PCIU, with a rate of 23% (4).

The ETT depth was affected by a number of factors, including variations in head position. During head flexion, the ETT tip had been observed to move toward the carnia, inducing endobronchial intubation and even hypoxia. On the contrary, the ETT tip moved toward the vocal cord in the head extension (5–11). However, in the previous studies, the distance of the ETT tip movement varied with different angles of head position. And fewer studies had addressed the impact of head rotation on the ETT depth.

According to human anatomy, the average angle of the head flexion is 45° while the maximum angle of extension is 70°. If the angle of head rotation is more than 60°, the blood vessels in the neck will be twisted (12, 13). Therefore, our study aimed to assess the effect of the head 45° extension, 45° flexion, 60° right rotation and 60° left rotation on the ETT depth in children.

## Materials and methods

### Ethical approval

This study protocol was approved by the Medical Ethics Committee of the First Affiliated Hospital of Shantou University Medical College (No. B-2021-001) and registration on www.chictr.org.cn (24/01/2021, ChiCTR2100042648). All patients participating in this study voluntarily signed an informed parental consent.

### Participants

This prospective observational study included pediatric subjects aged 4–12 years old under general orotracheal anesthesia in the First Affiliated Hospital of Shantou University Medical College from January 2021 to September 2021. Subjects with the following conditions were excluded: (1) emergency surgery; (2) patients with limited head movement; (3) patients with difficult airways and any known or suspected airway anomalies; (4) patients with serious cardiopulmonary diseases such as asthma and congenital heart disease.

### Anesthesia and data collection

**FIGURE 1:**
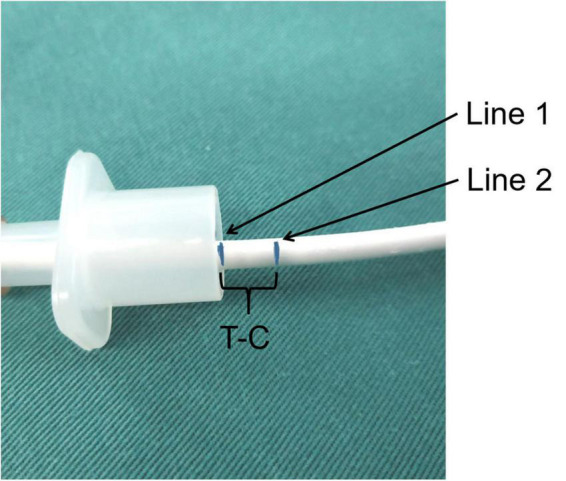
The intubation marker line during measuring. Line 1 represented that the tip of the endoscope arrived at the carina. Line 2 represented that the tip of the endoscope arrived at the tip of the ETT.

After admission to the operating room, routine monitoring of ECG, NIBP, SPO2, and ETCO2 were applied to each subject. General endotracheal anesthesia was induced with propofol (3 mg/kg), sufentanil (0.4 μg/kg), cisatracurium (0.2 mg/kg) IV. There were two ETT types: the taper guard oral tracheal tube (the taper guard ETT) (TaperGuard Oral/Nasal Tracheal Tube, Mruphy Eye, Covidien) and the reinforced oral tracheal tube (the reinforced ETT) (Lo-Contour Oral/Nasal Tracheal Tube Cuffed, Reinforced, Mruphy Eye, Covidien). Both catheters are pediatric tracheal tubes with large capacity low-pressure cuff made of ultra-thin polyurethane. And the ETT sizes were selected by the anesthesiologists according to the formula = age/4 + 3.5 from Pediatric Life Advanced Support (14). After induction, oral intubation was performed under direct laryngoscopy. An attending anesthesiologist opened the subject’s mouth by separating the lips and inserted the laryngoscope by the left hand into the mouth. Then advancing the laryngoscope blade slowly and pushing the tongue to the left side of the mouth to expose the uvula and epiglottis. Next the laryngoscope blade was lifted up to bring the vocal cord into view. The anesthesiologist then inserted the ETT through the right corner of the mouth into the trachea and the first intubation depth marker line (Figure 1) of the ETT was placed between the vocal cords (15, 16). The correct ETT position was confirmed by end-tidal carbon dioxide examination and lung auscultation. Dental pad was placed on the left side of the ETT. After the ETT was fixed, the anesthesiologists recorded the ETT depth at the upper incisor teeth. Anesthesia was maintained with continuous infusion of propofol of 9–15 mg/kg/h and remifentanil of 0.1–0.25 μg/kg/min according to BIS (maintained between 50 and 60). Ventilator parameters were set: tidal volume, 8–10 ml/kg; respiratory rate 15–25 times/min; I: E, 1:2; ETCO2 35–45 mmHg.

General information of all subjects was recorded during the operation, including ASA classification, age, gender, height, weight, BMI, the ETT types, the surgical types and perioperative adverse effects of endobronchial intubation, accidental tracheal extubation, hoarseness and sore throat.

### Measurement

**FIGURE 2:**
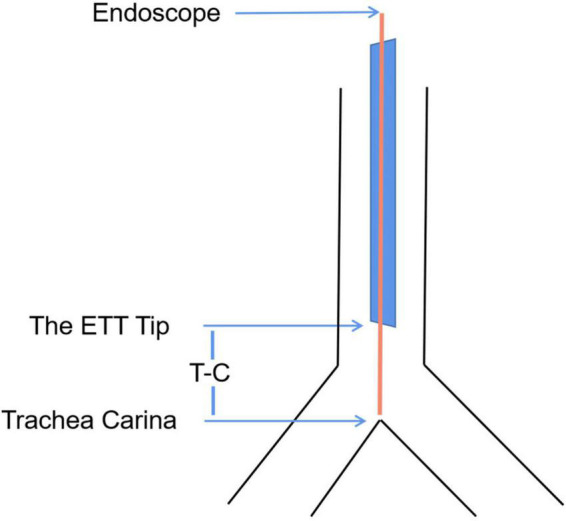
The distance between the ETT tip and the carina (T-C).

After endotracheal intubation, the subjects were in a supine position with head in a neutral position. The distance between the ETT tip and the carina was measured using the Disposcope flexible endoscope (Disposcope, Changsha, Hunan, China) by the following steps: (1) When the tip of the Disposcope flexible endoscope arrived at the carina, marking the first line at the intersection of the endoscope and the ETT. (2) The Disposcope flexible endoscope was then retracted till the end of the scope reached the tip of the ETT and the second line was marked. (3) The distance between the ETT tip and the carina (T-C) was corresponded to the distance between the two lines above (Figure 2). And T-C were measured using the same method in the following head positions: 45° extension, 45° flexion, 60° right rotation, and 60° left rotation. After removing the Disposcope flexible endoscope, all distances were measured using a caliper rule.

**FIGURE 3:**
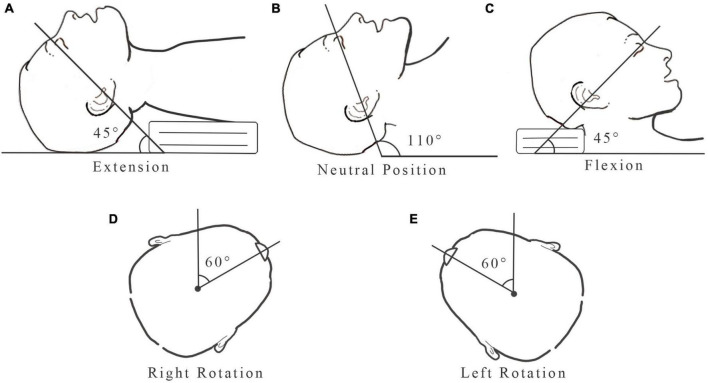
Head position: **(A)** 45° extension; **(B)** neutral position; **(C)** 45° flexion; **(D)** 60° right rotation; **(E)** 60° left rotation.

According to Boydin’s definition (17), the angle between the line from the superior orbital margin to the external auditory canal, relative to the horizontal line was 110° in head neutral position. The head was placed in extension position with the angle between the line from the superior orbital margin to the external auditory canal, relative to the horizontal line was 45° (a pillow was placed under the head). The head was placed in flexion position with the angle between the line from the superior orbital margin to the external auditory canal, relative to the horizontal line was 45° (a pillow was placed under the shoulder blades). A goniometer is used to measure the head rotation angle. The rotation angle between the line from the upper center of the head to the tip of the nose, relative to the vertical line was 60° (Figure 3).

The ETT was considered displaced when the distance of the ETT tip movement (ΔT-C) which was the difference between the T-C in the head neutral position and the other position was ≥ 0.5 cm. Positive ΔT-C indicated that the ETT moved toward the carina, whereas negative ΔT-C indicated that the ETT moved toward the vocal cord. The primary observation index was the distance and the direction of the ETT tip movement after changing head position. Secondary observation indicators were the perioperative adverse events.

### Statistical analysis

Sample size estimation was calculated by the PASS 15.0 (NCSS, Kaysville, UT, United States). A minimum of one hundred and eighteen subjects were required in order to achieve a power of 80% at the 0.05 level of significance and a dropout rate of 10%. Data was analyzed using SPSS 22.0 (IMB, Chicago, United States). Continuous variables, including age, height, weight, BMI and T-C were expressed as mean (SD), and categorical variables, including sex, surgical types and perioperative adverse events were expressed as frequency. Paired *t*-test was used to compare the differences of T-C when the head position changed from neutral position to 45° extension, 45° flexion, 60° right and left rotation. Pearson correlation analysis was used to examine the correlation between age, gender, height, weight, BMI and the ETT types and the distance of the ETT tip movement. The correlation was expressed as the *r*-value which was indicated: *r* < 0.4, weak correlation; 0.4 ≤ *r* < 0.7, moderate correlation; *r* ≥ 0.7, strong correlation. A value of *P* < 0.05 was considered significant.

## Results

One hundred and eighteen subjects undergoing elective surgeries were included in this study. And six subjects were excluded, including five subjects with decreased SpO_2_ due to prolonged measurement time and one subject with increased airway pressure above 30 mmHg after intubation. One hundred and twelve pediatric subjects were finally included (Tables 1, 2).

**TABLE 1:** Characteristics of 112 subjects.

Characteristics	Total
*N*	112
Years (y)	7 ± 2 (4–12)
Gender (M/F)	87/25
Height (cm)	123.7 ± 15.1 (99.0–168.0)
Weight (kg)	25.4 ± 10.2 (13.0–67.0)
BMI (kg/m^2^)	16.0 ± 3.5 (9.6–28.3)

**TABLE 2:** Characteristics of 112 subjects based on age grouping.

Year	*N*	Sex (M/F)	Height (cm)	Weight (kg)	BMI	ASA (I/II)
4	19	18/1	103.9 ± 2.5	16.2 ± 1.6	15.0 ± 1.4	19/0
5	13	8/5	112.4 ± 3.1	18.7 ± 1.7	14.7 ± 0.9	13/0
6	16	12/4	116.0 ± 5.3	21.4 ± 3.7	15.8 ± 2.0	16/0
7	16	11/5	125.6 ± 7.0	25.0 ± 5.7	15.6 ± 2.2	16/0
8	14	11/3	127.5 ± 6.7	27.2 ± 10.6	15.6 ± 6.2	14/0
9	9	7/2	134.8 ± 6.3	27.1 ± 3.9	14.9 ± 1.2	9/0
10	16	13/3	139.6 ± 10.5	35.6 ± 9.7	18.1 ± 3.7	16/0
11	5	4/1	141.0 ± 3.5	32.2 ± 10.4	16.1 ± 5.0	5/0
12	4	3/1	154.2 ± 12.8	49.9 ± 14.5	21.0 ± 5.8	4/0

### The distance and the direction of the endotracheal tube tip movement

**FIGURE 4:**
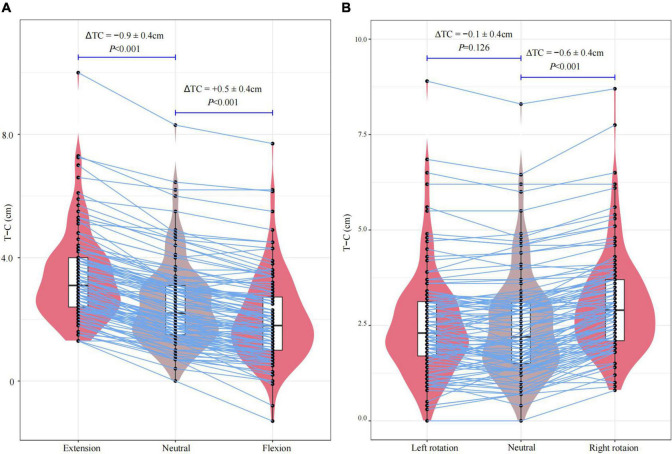
The distance of the ETT tip movement after changing head position: **(A)** 45° extension and 45° flexion from the neutral position; **(B)** 60° right rotation and 60° left rotation from the neutral position.

The distance of the ETT tip movement in different head position were shown in Tables 3, 4 and Figure 4. The ETT tip moved toward the vocal cords by 0.9 ± 0.4 cm (*P* < 0.001) with head 45° extension and toward the carina by 0.5 ± 0.4 cm (*P* < 0.001) with head 45° flexion. During head-neck rotation to the right, the ETT tip moved toward the vocal cords by 0.6 ± 0.4 cm (*P* < 0.001). There was no displacement of the ETT tip in head 60° left rotation and the direction of the ETT tip movement could not be predicted (*P* = 0.126).

**TABLE 3:** The distance of the ETT tip movement.

	T-C (cm)	Δ T-C (cm)	*P*
Neutral	2.5 ± 1.4		
Left rotation	2.6 ± 1.4	–0.1 ± 0.4	0.126
Right rotation	3.1 ± 1.3	–0.6 ± 0.4	<0.001
Flexion	2.0 ± 1.5	+ 0.5 ± 0.4	<0.001
Extension	3.4 ± 1.5	–0.9 ± 0.4	<0.001

**TABLE 4:** The direction of the ETT tip movement.

	To the carina	To the vocal cord	No displacement
Left rotation	53 (47.3%)	42 (37.5%)	17 (15.2%)
Right rotation	4 (3.6%)	97 (86.6%)	11 (9.8%)
Flexion	94 (83.9%)	7 (6.3%)	11 (9.3%)
Extension	0 (0%)	108 (96.4%)	4 (3.6%)

### Pearson correlation analysis of the distance of the endotracheal tube tip movement

Pearson correlation analysis showed weak positive correlations between the distance of the ETT tip movement and age, height, weight and BMI at head 60° left rotation. When the head position was in 45° flexion, the distance of the ETT tip movement was weakly negatively correlated with age and height, and weakly positively correlated with sex. However, no correlation between the distance of the ETT tip movement and the five biological factors was found in head 45° extension and 60° right rotation. Moreover, the ETT types had weak positive correlations with the distance of the ETT tip movement in head right rotation and extension. No correlation was observed between the distance of the ETT tip movement and the ETT types in head left rotation and flexion (Table 5).

**TABLE 5:** Correlation of the distance of the ETT tip movement (r).

Head position	Age	Gender	Height	Weight	BMI	ETT types
Left rotation	0.300**	–0.045	0.231*	0.243**	0.203*	0.149
Right rotation	0.078	–0.073	0.066	0.130	0.183	0.268**
Flexion	–0.251**	0.219*	–0.207*	–0.109	0.005	–0.058
Extension	0.083	–0.094	0.061	–0.034	–0.094	0.268**

“*” and “**,” respectively, indicate significant correlation at the P < 0.05 level and P < 0.01 level.

For further analysis, in head 60° right rotation, the distance of the reinforced ETT tip movement was more (0.6 ± 0.4 cm) than the taper guard ETT (0.4 ± 0.4 cm) (*P* = 0.001). in head 45° extension, the distance of the reinforced ETT tip movement was more (1.0 ± 0.4 cm) than the taper guard ETT (0.7 ± 0.4 cm) (*P* = 0.004). There was no statistical difference in the magnitude of the ETT tip displacement between the two groups in the head left rotation (*P* = 0.080) and flexion (*P* = 0.545) (Table 6).

**TABLE 6:** Magnitude of the ETT tip movement grouped by the ETT types.

	Taper guard ETT	Reinforced ETT	*P*
Left rotation (cm)	+ 0.1 ± 0.4	+0.0 ± 0.3	0.080
Right rotation (cm)	+ 0.6 ± 0.4	+0.4 ± 0.4	0.001
Flexion (cm)	–0.5 ± 0.4	–0.4 ± 0.4	0.545
Extension (cm)	+ 1.0 ± 0.4	+0.7 ± 0.4	0.004

### Adverse event

Endobronchial intubation occurred in two subjects with the head position in 45° flexion. There was no accidental tracheal extubation, hoarseness or sore throat in all 112 subjects.

## Discussion

Because of their short trachea, precise depth of tracheal tube insertion is mandatory in children in order to avoid either inadvertent extubation or endobronchial intubation during head-neck movement. Head-neck extension, flexion and rotation during anesthesia in tracheally intubated children is common during ENT surgery, dental or cleft surgery as well as neurosurgery for posterior fossa craniotomies, whereas the effects of rotation are unpredictable.

This study has indicated that the head movements have effects on the position of the ETT in 4–12-years old children. The ETT tip moved toward the vocal cord when the head-neck position was in extension and right rotation, while moved toward the carina in head flexion. In head left rotation, there is no ETT tip movement.

The clinically difference in our study was based on the surgical anatomy of the anterior branch of the recurrent laryngeal nerve. Previous studies have pointed out that the anterior branch of the recurrent laryngeal nerve was positioned about 6–10 mm beneath the vocal cords in adults and would be frail compressed by the cuff (18, 19). And the safe range of the ETT position was narrow in children than in adults because of the short trachea length. Thus, the study determined that the minimal clinically difference (ΔT-C) was 0.5 cm.

Yan and Zhang (11) reported that the average distance of the ETT tip movement in 2–12-years-old children was 1 cm in both the maximum flexion and sniffing position. Kim et al. (7) explored the effect of head rotation, flexion, and extension on the ETT position in 22 pediatric subjects aged 1–9 years. The results suggested that the mean distance of the ETT tip movement was more than 1 cm when the head positions were in flexion, extension, and right rotation, while the angle of the head positions changes was not mentioned in the study. In contrast to the previous studies (7, 11), the average distance of the ETT tip movement in the head 45° flexion in our study was 0.5 ± 0.4 cm. We speculate that the reason for the difference of the distance was caused by the fact that the head flexion angle in our study was 45°, which was less than the maximum flexion angle in Yan and Zhang (11) study. We found that in 83.9% (94/112) of cases, the ETT tip moved toward the carina during head 45° flexion and endobronchial intubations were noted in two subjects. These adverse events showed that inappropriate ETT depth caused by changes in head position may lead to unpredictable intubation complications. While in 6.3% (7/112) of cases, the tip moved toward the vocal cord instead of the carina. Similarly, Tailleur et al. (6) reported that there was no fixed direction of the ETT movement after head flexion and there were 24% of subjects showed displacement of the ETT tip toward the vocal cord. We concluded that the direction of the ETT movement due to head flexion was unpredictable. It was also critical to confirm the ETT depth after shifting the head position to prevent intubation complications.

Our study indicated that when the head position was in 45° extension, the mean movement was 0.9 ± 0.4 cm. Moreover, the ETT tip was displaced in the direction of the vocal cord which was similar to the results of the previous studies (7, 8, 10, 11). The previous study also demonstrated that when the head in extension position, the distance of the ETT tip movement was more than the expanded distance of the airway length (20). The risk of accidental tracheal extubation of the ETT remained when the head was in sniffing position. To expose the surgical area, the patient’s head position was commonly changed to extension during otolaryngology surgeries. As a result, we recommend that the ETT depth of head extension be 1 cm greater than in the supine position. Additionally, the ETT position must be confirm using bilateral lung auscultation and PetCO2.

Although the angle of the head rotation was the same, we found that the distance of the ETT tip movement was greater in the head right rotation than the left. We speculated that the discrepancy of the distance between the right and left rotation was related to the dental pad placement. In our study, the dental pad was placed on the left side of the ETT, implying that the ETT was fastened on the right side of the mouth. When the head was rotated to the fixed side, the distance of the ETT tip movement was greater than that of the opposite side. The result is similar to the study by Kim et al. (7) and Kim (9). However, the distance of the ETT tip movement in the head right rotation in our study was lower which was related to the smaller rotation angles. The children’s head were frequently turned to either side to get a better view of the operative region during cochlear implantation or buccal surgery. To minimize the ETT displacement, we recommend placing the ETT in the corner of the mouth on the same side as the surgical site. The direction of the ETT tip movement after head rotation remained controversial in previous studies. Kim et al. (7) reported that the ETT tip moved toward the vocal cord when the head rotated to either side which was similar to the results of our study. While the direction of the ETT tip movement was unpredictable after maximal head movements in adults (6). Further studies were needed to explore whether the direction of the ETT tip movement in pediatric patients is the same as that in adults.

In addition, weak correlation implied less effect of biological factors such as age, gender, height, weight and BMI on the distance of the ETT tip movement. After adjusting the head position, the distance of the ETT tip movement varied depending on the type of the ETT. The tip of the reinforced ETT moved less than the taper guard ETT when the head positions were in 60° right rotation and 45° extension. The reinforced ETT was equipped with a spiral hose on the inner wall and a spring inside the spiral hose, which made the tube soft and flexible, as well as resistant to bending and compression. The trachea was a cylindrical tube, supported by “C” shaped tracheal cartilage, connected by a cricoid ligament, and surrounded by smooth muscle tissue and fibrous connective tissue to form a membranous wall (5). The trachea’s anatomical characteristics allowed it to be flexible enough to accommodate the head movements. Toung et al. used computed tomography to demonstrate that the tracheal length reduced when the head were flexed and increased when the head were extended (21, 22). Therefore, the reinforced ETT had more substantial ductility and less displacement of the ETT tip after changing the head position, resulting in better safety. We recommend the use of the reinforced ETT for children in otolaryngology surgery or cranial surgeries.

This study had the following limitations: Firstly, infants were not included in this study. The younger the child, the shorter the tracheal length. Intubation complications, such as bronchial intubation or accidental extubation were more common in infants than in older children. Further studies were needed to investigate the effect of head position changes on the depth of endotracheal intubation in infants. Secondly, the study did not investigate the displacement of the ETT tip movement following other head position adjustment. A further limitation of the study was that only children who were orally intubated were examined. However, Hartrey and Kestin (23) discovered no difference in ETT displacement between oral and nasal intubations during head extension or flexion in adults. Another study (5) reported that the trachea length and the distance between the nares and the vocal cords were both changed during head extension or flexion in children. And the displacement of the ETT depended mainly on the changes in trachea length. As a result, further investigation is necessary to compare the magnitude of the ETT displacement during nasal tracheal and oral tracheal intubation after changing head positions in children.

## Conclusion

The ETT tip moved toward the vocal cord in head 45° extension in pediatric patients, and when the head positions were in 45° flexion, the ETT tip moved toward the carina. When the head rotation to the right, the ETT moved toward the glottis, and there was no substantial displacement in 60° left rotation. The ETT depth was less impacted by head position changes in subjects with a reinforced tracheal tube than with a taper-guard oral tracheal tube. Therefore, we recommend the use of a reinforced tracheal tube in pediatric surgery.

## Data availability statement

The raw data supporting the conclusions of this article will be made available by the authors, without undue reservation.

## Ethics statement

The studies involving human participants were reviewed and approved by the Medical Ethics Committee of The First Affiliated Hospital of Shantou University Medical College. Written informed consent to participate in this study was provided by the participants’ legal guardian/next of kin.

## Author contributions

MC and PZ contributed to design of the study. PZ collected the data and wrote the manuscript. WW finished the statistical analysis and revised the manuscript. MC and WW checked the data and finished the project administration. All authors contributed to manuscript revision, read, and approved the submitted version.

## References

[1] Morgan GA Steward DJ. Linear airway dimensions in children: including those from cleft palate. canadian anaesthetists’. *Soc J.* (1982) 29:1–8. 10.1007/BF03007939 7055739

[2] Owen RL Cheney FW. Endobronchial intubation: a preventable complication. *Anesthesiology.* (1987) 67:255–7. 3605754 10.1097/00000542-198708000-00019

[3] Matsuoka W Ide K Matsudo T Kobayashi T Nishimura N Nakagawa S. The occurrence and risk factors of inappropriately deep tip position of microcuff pediatric endotracheal tube during PICU Stay: a retrospective cohort pilot study. *Pediatr crit Care Med.* (2019) 20:e510–5. 10.1097/PCC.0000000000002097 31517729

[4] Rivera R Tibballs J. Complications of endotracheal intubation and mechanical ventilation in infants and children. *Survey Anesthesiol.* (1992) 36:193–9. 10.1097/00003246-199202000-00008 1737455

[5] Yamanaka H Tsukamoto M Hitosugi T Yokoyama T. Changes in nasotracheal tube depth in response to head and neck movement in children. *Acta Anaesthesiol Scand.* (2018) 62:1383–8. 10.1111/aas.13207 29971764

[6] Tailleur R Bathory I Dolci M Frascarolo P Kern C Schoettker P. Endotracheal tube displacement during head and neck movements. Observational clinical trial. *J Clin Anesthesia.* (2016) 32:54–8. 10.1016/j.jclinane.2015.12.043 27290945

[7] Kim JT Kim HJ Ahn W Kim HS Bahk JH Lee SC Head rotation, flexion, and extension alter endotracheal tube position in adults and children. *Canad J Anaesth.* (2009) 56:751–6. 10.1007/s12630-009-9158-y 19639372

[8] Weiss M Knirsch W Kretschmar O Dullenkopf A Tomaske M Balmer C Tracheal tube-tip displacement in children during head-neck movement–a radiological assessment. *Br J Anaesth.* (2006) 96:486–91. 10.1093/bja/ael014 16464981

[9] Kim S. Comparison of the cuff pressures of a TaperGuard endotracheal tube during ipsilateral and contralateral rotation of the head: A randomized prospective study. *Medicine.* (2018) 97:e12702. 10.1097/MD.0000000000012702 30334954 PMC6211887

[10] Jordi Ritz EM Von Ungern-Sternberg BS Keller K Frei FJ Erb TO. The impact of head position on the cuff and tube tip position of preformed oral tracheal tubes in young children. *Anaesth.* (2008) 63:604–9. 10.1111/j.1365-2044.2008.05440.x 18477271

[11] Yan S Zhang H. Impact of changes in head position during head and neck surgery on the depth of tracheal tube intubation in anesthetized children. *BMC Anesthesiol.* (2020) 20:124. 10.1186/s12871-020-01033-7 32448244 PMC7245884

[12] Thompson JC Netter FH. *Netter’s. Concise Orthopaedic Anatomy*. Philadelphia: Icon Learning Systems (2010).

[13] Norkin CC White DJ. *Measurement of Joint Motion: A Guide to Goniometry.* Philadelphia, PA: F.A. Davis (2003).

[14] Topjian AA Raymond TT Atkins D Chan M Duff JP Joyner BL Jr. Part 4: pediatric basic and advanced life support: 2020 american heart association guidelines for cardiopulmonary resuscitation and emergency cardiovascular care. *Circulation.* (2020) 142 16(Suppl 2):S469–523. 33081526 10.1161/CIR.0000000000000901

[15] Weiss M Balmer C Dullenkopf A Knirsch W Gerber A Bauersfeld U Intubation depth markings allow an improved positioning of endotracheal tubes in children. *Canad J Anaesth.* (2005) 52:721–6. 10.1007/BF03016560 16103385

[16] Chong DY Greenland KB Tan ST Irwin MG Hung CT. The clinical implication of the vocal cords-carina distance in anaesthetized Chinese adults during orotracheal intubation. *Br J Anaesth.* (2006) 97:489–95. 10.1093/bja/ael186 16873383

[17] Boidin MP. Airway patency in the unconscious patient. *Br J Anaesth.* (1985) 57:306–10. 3978013 10.1093/bja/57.3.306

[18] Kim H Chang JE Ryu JH Jung H Min SW Lee JM Retrospective analysis of vocal cord-to-suprasternal notch distance: Implications for preventing endotracheal tube cuff-induced vocal cord injury. *Medicine (Baltimore).* (2017) 96:e6155. 10.1097/MD.0000000000006155 28207550 PMC5319539

[19] Cavo JW Jr. True vocal cord paralysis following intubation. *Laryngoscope.* (1985) 95:1352–9. 4058215 10.1288/00005537-198511000-00012

[20] Jin-Hee K Ro YJ Seong-Won M Chong-Soo K Seong-Deok K Lee JH Elongation of the trachea during neck extension in children: implications of the safety of endotracheal tubes. *Anesth Analg.* (2005) 101:974-7. 10.1213/01.ane.0000169330.92707.1e 16192505

[21] Penning L. Radioanatomy of upper airways in flexion and retroflexion of the neck. *Neuroradiology.* (1988) 30:17–21. 10.1007/BF00341937 3357563

[22] Toung TJ Grayson R Saklad J Wang H. Movement of the distal end of the endotracheal tube during flexion and extension of the neck. *Anesth Analg.* (1985) 64:1030–2. 4037385

[23] Hartrey R Kestin IG. Movement of oral and nasal tracheal tubes as a result of changes in head and neck position. *Anaesthesia.* (1995) 50:682–7. 10.1111/j.1365-2044.1995.tb06093.x 7645696

